# Validation of the German version of the Neck Disability Index (NDI)

**DOI:** 10.1186/1471-2474-15-91

**Published:** 2014-03-19

**Authors:** Holger Cramer, Romy Lauche, Jost Langhorst, Gustav J Dobos, Andreas Michalsen

**Affiliations:** 1 Department of Internal and Integrative Medicine, Kliniken Essen-Mitte, Faculty of Medicine, University of Duisburg-Essen, Am Deimelsberg 34a, 45276 Essen, Germany; 2 Department of Internal and Integrative Medicine, Immanuel Hospital Berlin, Königstraße 63, 14109 Berlin, Germany; 3 Institute for Social Medicine, Epidemiology, and Health Economics, Charité University Medical Center, Königstraße 63, 14109 Berlin, Germany

**Keywords:** Neck pain, Chronic pain, Disability, Neck disability index, Translation, German, Validity, Reliability, Factor structure, Sensitivity to change

## Abstract

**Background:**

The Neck Disability Index (NDI) is the most commonly used outcome measure for neck pain. This study aimed to determine the psychometric properties of a German version of the NDI. Cross-cultural translation and psychometric testing of the NDI were performed.

**Methods:**

The 10-item NDI was translated into German and administered to 558 patients with chronic unspecific neck pain (Mean age 49.9 ± 11.4 years, 76% female). The factor structure and reliability of the NDI were assessed using factor analysis, Cronbach’s alpha, split-half reliability (Spearman-Brown coefficient), and intra-class correlation (ICC_2,1_). To determine convergent validity, pain intensity (visual analog scale; VAS), pain on movement (VAS), and quality of life (Short Form 36 Health Survey Questionnaire; SF-36) were correlated with the NDI. Correlation with range of motion and sensitivity to change were also assessed in a subsample of 49 patients.

**Results:**

The mean NDI score was 32.75 ± 13.09. Factor analysis revealed a single factor that explained 39.8% of the variance. Cronbach’s alpha was 0.81; Spearman-Brown coefficient was 0.80; and intra-class correlation was 0.81 (95% confidence interval = 0.78, 0.83). Significant correlations were found for pain intensity (r = 0.22, p < 0.01), pain on movement (r = 0.39, p < 0.01), quality of life (r = -0.30 to -0.45, p < 0.01), and range of motion (r = -0.34, p = 0.02). Patients who reported global improvement of health after an exercise or yoga intervention showed a higher decrease on the NDI than patients who reported no global improvement (p < 0.01).

**Conclusions:**

The German version of the NDI has a comparable factor structure as the original version, acceptable psychometric properties, and is sensitive to change after physical activity. Neck disability is associated with other measures of neck pain.

## Background

With a mean lifetime prevalence of about 50%, neck pain is a major public health problem in all industrialized countries [1]. Despite its often unclear physiological correlates and episodic nature, neck pain is often associated with marked disability: more than 10% of work absenteeism is at least somehow associated with neck pain [2], and about 5% of adults report significantly disabling neck pain [3]. Therefore, the assessment of neck pain and its progression is mostly based on patient-based outcome measures, mainly pain intensity and disability [4]. The Neck Disability Index is probably the most commonly used and best validated instrument for assessing disability in patients with neck pain [5]. Originally based on the Oswestry Low Back Pain Index, the Neck Disability Index assesses neck-related disability on 10 items [6]. The internal consistency generally is reported to be high for both the original [6] and translated versions of the instrument [7-11]. While most validation studies have replicated the original single factor structure [7,9], other studies found a better fit of the data with a two factor solution [10,11]. The validity of the Neck Disability Index has also been demonstrated for a number of translations and cross-cultural adaptations including the Japanese, Portuguese, Spanish, and Swedish version [12]. However, the only available German version of the instrument [13] has been criticized for the extremely small sample size in its validation study that strongly limits the usefulness of this version [14].

Therefore, this analysis of 558 patients suffering from chronic non-specific neck pain aimed to determine reliability and validity of a German version of the Neck Disability Index.

## Methods

### Subjects

Patients from 10 randomized controlled trials on chronic non-specific neck pain were included in psychometric testing. The trials had been conducted at the Department of Complementary and Integrative Medicine, Kliniken Essen-Mitte, Germany [15-22] and at the Immanuel Hospital Berlin, Department of Internal and Integrative Medicine, Berlin, Germany [23,24]. All studies were conducted in accordance with the ethical standards of the responsible institutional committee on human experimentation and with the Helsinki Declaration of 1975, as revised in 1983. All studies were approved by the institutional ethics committee of the medical institutions at the University of Duisburg-Essen, Germany [15-22] or by the Ethics Committee of the Charité-University Medical Center, Berlin, Germany [23,24] prior to patient recruitment and all patients gave written informed consent prior to inclusion in the study.

To be included, adult patients of both genders had to suffer from chronic non-specific neck pain for at least 5 days a week for at least 12 consecutive weeks with a mean pain intensity of at least 40 mm on a 100 mm visual analog scale (VAS) from 0 mm (no pain) to 100 mm (worst pain imaginable). Patients with neck pain that was likely to result from specific causes such as neck trauma, disc protrusion, rheumatic diseases, spinal canal stenosis, or neoplasms as well as patients with serious physical or mental comorbidities such as active oncologic diseases, diabetes mellitus, coronary artery disease, affective disorders, addiction, or psychosis were excluded.

#### Sample description

The psychometric sample consisted of 558 patients of which 134 (24.0%) were male and 424 (76.0%) were female. Age ranged from 19 to 81 years with a mean age 49.9 ± 11.4 years. The mean pain intensity was 45.7 mm ± 20.8 mm on a VAS; mean pain duration was 7.54 ± 7.52 years (Table 1).

**Table 1:** Sociodemographic and clinical characteristics of the psychometric sample (mean ± standard deviation)

**Variable**	**Total (n = 458)**	**Responsiveness subsample (n = 49)**
**Gender: N female (%)**	424 (76%)	40 (81.6%)
**Age (in years)**	49.89 ± 11.40	47.92 ± 10.59
**Pain intensity (in mm VAS)**	45.69 ± 20.77	44.33 ± 19.02
**Pain duration (in years)**	7.54 ± 7.52	8.01 ± 6.38
**Neck disability index**	32.75 ± 13.09	27.36 ± 9.42
**Pain on movement (in mm VAS)**	42.85 ± 20.52	40.76 ± 20.07
**Health-related quality of life (SF-36)**		
Physical functioning	75.32 ± 17.19	80.59 ± 14.48
Physical role functioning	44.14 ± 37.64	53.65 ± 34.97
Bodily pain	42.13 ± 13.87	46.82 ± 10.98
General health perceptions	60.05 ± 18.64	61.10 ± 19.04
Vitality	47.78 ± 18.58	47.55 ± 16.77
Social functioning	70.38 ± 23.58	72.96 ± 21.09
Emotional role functioning	64.61 ± 41.04	66.67 ± 39.51
Mental health	62.13 ± 17.62	64.41 ± 16.23
Physical component score	40.85 ± 7.93	43.21 ± 8.01
Mental component score	45.09 ± 11.63	45.28 ± 11.01
**Range of motion**	-	45.52 ± 9.04

*Abbreviations*: SF-36 Short form 36 health survey, VAS visual analog scale.

### Measures

#### Neck-specific disability

As a measure of neck-specific functional disability, a translated version of the original 10-item Neck Disability Index (NDI) was used [6]. The NDI covers 10 dimensions of neck-specific disability, namely pain intensity, personal care, lifting, reading, headache, concentration, work, driving, sleeping, and recreation [6]. Each dimension is assessed with 1 item, measured on a 6-point scale from 0 (no disability) to 5 (full disability). The sum score out of all 10 items is multiplied by 2 to obtain a score out of 100% [12]. The instrument was translated into German independently by 2 German native speakers with intensive English language training and knowledge of the English-speaking culture. Both translators were health professionals and experienced in assessing questionnaire and interview data. The translation aimed at a conceptual equivalent of the item rather than a word-for-word translation. Both translations were combined by the translators into a single consensus translation by discussion. The instrument was then back-translated into English by 2 independent professional translators who had no knowledge of the original instrument and had no health back ground. Again, a single back-translation was produced by discussion until consensus was reached. Concordance of the back-translated version and the original NDI was approved by the senior author (AM). The final German version of the NDI is given in Additional file 1.

#### Neck pain intensity

Mean neck pain intensity during the past 7 days was measured on a 100-mm VAS [25].

#### Pain on movement

Pain on movement was assessed for 6 consecutive head movements (flexion, extension, lateral flexion left/right, rotation left/right). The pain elicited by each of these movements was scored on a 100-mm VAS [18].

#### Health-related quality of life

Health-related quality of life was assessed using the Short Form 36 Health Survey Questionnaire (SF-36) [26]. This comprehensive 36-item questionnaire yields an 8-scale health profile as well as two component summaries of physical and mental health-related quality of life.

#### Cervical range of motion

In a subsample of 49 patients, cervical range of motion was measured for 6 cervical movement directions (flexion, extension, lateral flexion left/right, rotation left/right) using an electromagnetic, motion-tracking device (Fastrak, Polhemus, Colchester, USA) [19,27]. A small sensor was attached to the forehead using a Velcro strap and movements of this sensor were converted to Euler angles. Patients were asked to move their head as far as possible at a comfortable speed. Three trials for each movement direction were performed and averaged.

### Statistical analysis

#### Construct validity

Given that prior cross-cultural adaptations of the NDI had revealed either single factor or two factor solutions, no a priori assumptions about the underlying factor structure of the German NDI were made and an exploratory factor analysis using the principle components extraction and the varimax rotation was performed on the 10 items of the NDI to explore the instrument’s structure. Factors were extracted if their eigenvalue was >1. Domain scores of any resulting factors, or of a total score, were calculated as a sum of the component item scores. A major requirement for factor analysis is a considerably large subject:item ratio. A minimum ratio of 20:1 has been proposed to ensure strong factor structures are replicated reliably [28]. This requirement was met by the subject:item ratio of 1:55.8 in this analysis.

#### Reliability

To assess internal consistency of the NDI, Cronbach’s α, alpha if item deleted, item-scale correlations, and item difficulty were calculated. Split-half reliability was assessed as the Spearman-Brown coefficient for the first 5 items (pain intensity, personal care, lifting, reading, headache) and the last 5 items (concentration, work, driving, sleeping, and recreation). Two-way random intra-class correlation (ICC_2,1_) and its 95% confidence interval (CI) was used to assess agreement between measures.

#### Convergent validity

As a measure of convergent validity, the strength of relationship of the NDI with theoretically related instruments for neck pain patients was assessed. Spearman’s correlation coefficients between the NDI, pain intensity, pain on movement, and the component summaries and subscales of the SF-36 were calculated. Correlation coefficient was further calculated for range of motion in a subsample of 49 patients.

#### Responsiveness

Sensitivity to change was calculated in a subsample of 49 patients. After the first assessment, those patients participated in a 9-week exercise or yoga intervention. The intervention consisted of 15 minutes of self-directed daily home practice based on manualized instructions and (for the yoga intervention) weekly 90-minute classes with a certified instructor [19]. After the intervention, the assessment was repeated. Additionally, global improvement was assessed on a 5-point Likert-type scale ranging from "my health is much better" to "my health is much worse" and patients were divided into two groups comprising those with stable vs. those with improved health. NDI change scores between the two groups were compared using an unpaired t-test.

All statistics were performed using the statistical package IBM SPSS Statistics (Version 20.0; IBM Inc., New York, USA). A p-value of <0.05 (two-tailed) was considered significant in all analyses.

## Results

### Descriptive scale characteristics

Mean item values ranged from 0.45 to 2.24 (Table 2). For all item but "personal care", the whole 0–5 range of the item was scored by at least 1 patient. For "personal care", the highest score was 3 ("I need some help but can manage most of my personal care"); 99.3% of the patients scored 2 or lower. The total instrument had a mean of 32.75 with a standard deviation of 13.09, values ranged from 8.00 (n = 4, 0.7%) to 82.00 (n = 1; 0.2%).

**Table 2:** Descriptive scale characteristics, factor structure, and reliability of the German version of the Neck Disability Index (NDI)

**Item**	**Mean ± SD**	**Item difficulty**	**Factor loading**	**Corrected item-total correlation**	**Alpha if item deleted**
Pain intensity	2.24 ± 0.77	0.45	0.57	0.44	0.80
Personal care	0.45 ± 0.63	0.09	0.67	0.54	0.80
Lifting	1.53 ± 1.12	0.31	0.56	0.42	0.80
Reading	2.07 ± 1.08	0.41	0.61	0.52	0.80
Headache	2.17 ± 1.38	0.43	0.48	0.38	0.81
Concentration	1.15 ± 1.04	0.23	0.71	0.59	0.78
Work	1.14 ± 1.00	0.23	0.76	0.63	0.78
Driving	2.05 ± 1.32	0.41	0.55	0.44	0.80
Sleeping	1.64 ± 1.26	0.33	0.59	0.47	0.79
Recreation	1.67 ± 0.86	0.33	0.75	0.64	0.78

### Factor structure of the NDI

Kaiser-Meyer-Olkin’s measure of sampling adequacy was satisfactory with 0.89, indicating that the sample was suitable for factor analysis. Primary component factor analysis revealed a single factor structure which would explain 39.8% of the variance (Table 2). Factor loadings ranged from 0.484 to 0.755 (Table 2). While the item "headache" had a factor loading < 0.5, the removal of this item from the analysis did not change Cronbach’s α. Therefore, it was decided to retain the item in the instrument to ensure consistency with the original English language instrument.

### Reliability

As a measure of internal consistency, Cronbach’s α was 0.81. For all items, deletion of the specific item would have reduced Cronbach’s α except for the item "headache" which deletion would not have changed alpha (Table 2). Corrected item total correlation ranged from 0.38 to 0.63; item difficulty ranged from 0.23 to 0.45 except for "personal care". "Personal care" had an item difficulty of 0.09 reflecting a generally low scoring. As the removal of this item from the analysis would have reduced Cronbach’s Alpha, it was decided to retain the item in the instrument. Testing for split-half reliability revealed a Spearman-Brown coefficient of 0.80. Intra-class correlation was ICC_2,1_ = 0.81, 95% CI = 0.78 to 0.83.

### Convergent validity

Results of the correlation analyses to determine the convergent validity of the NDI are shown in Table 3. NDI was significantly correlated with all measures. Correlations effect sizes were moderate for all measures except for pain intensity and emotional role function whose correlations with NDI had small effect sizes.

**Table 3:** Correlations of the neck disability index with pain intensity, pain on movement, health-related quality of life, and range of motion (in a subsample of n = 49)

**Variable**	**Neck disability index**	
	**Spearman’s ρ**	**p**
**Pain intensity**	0.22	<0.01
**Pain on movement**	0.39	<0.01
**Health-related quality of life (SF-36)**		
Physical functioning	-0.40	<0.01
Physical role functioning	-0.45	<0.01
Bodily pain	-0.44	<0.01
General health perceptions	-0.40	<0.01
Vitality	-0.45	<0.01
Social functioning	-0.42	<0.01
Emotional role functioning	-0.30	<0.01
Mental health	-0.36	<0.01
Physical component score	-0.43	<0.01
Mental component score	-0.32	<0.01
**Range of motion (n = 49)**	-0.34	0.02

### Responsiveness

Patients who reported global improvement of health after exercise or yoga showed a decrease of 7.04 ± 6.33 on the NDI; patients who reported no global improvement had a decrease of 0.82 ± 8.25; the group difference was significant (p < 0.01).

## Discussion

The German translation of the Neck Disability Index demonstrated a single factor structure with good internal consistency, intra-class correlation, and split-half reliability. The original English version was developed as a one-factor instrument [6]. The single factor solution has been supported in cross-cultural adaptations of Spanish [9], Brazilian/Portuguese [7], Greek [8], Finnish [29], and Turkish [30] translations. However, 2-factor structures were also found in a number of translated versions including the Italian [10], Polish [31], Arabic [32], and Japanese [11] versions. Recently, an alternative German version of the NDI has been validated in a total of 51 neck pain patients and healthy subjects [13]. While this validation study revealed a 2-factor solution, the interpretability of this analysis and hence the usefulness of the instrument is strongly limited by the extremely small sample size [14]. Compared to other versions of the Neck Disability Index, the explained variance of the single factor was relatively low in the German version reported here [12] but comparable to that of the Greek version [8]. Item-difficulty was relatively low for all items, especially for "personal care" that had not been scored higher than 3 out of 5 by any patient. More than a characteristic of the instrument, this might reflect a characteristic of the sample that reported only moderate pain intensity and relatively low disability despite the long duration neck pain. Strong disability in personal care might reflect a higher grade of general disability and have a higher impact on activities of daily living than e.g. disability in lifting. However, patients with Whiplash-associated disorder with more pronounced general disability have been shown to have higher personal care-related disability [33]. Despite the very low item difficulty of "personal care" and the low factor loading of "headache", removing these items would have reduced content validity of the instrument and make cross-cultural comparisons harder. Therefore, it was decided to retain the original 10-item structure.

Consistent with findings on other versions, the instrument was positively correlated with pain intensity and negatively with quality of life and neck range of motion [33-36]. This might reflect well-known associations between pain intensity, subjective disability, quality of life, and objective range of motion [37-39]. Interestingly, neck disability was associated not only with physical quality of life but also with all dimensions of mental quality of life. This is in line with earlier findings of correlation of the neck disability and mental health [40].

The instrument was sensitive to change after exercise or yoga interventions. Global impression of improved health was associated with improved neck disability. Several randomized trials have shown that exercise can improve global health and neck disability in chronic neck pain patients [41,42]. This also seems to apply to yoga intervention [19,23,43]. The German version of the Neck Disability Index seems to be responsive to these improvements. Responsiveness of a German version of the NDI has also been assessed in another study [44]. This study concluded that responsiveness to change was lower in the NDI than in the Bournemouth Questionnaire for Neck Pain. However, it remains unclear whether a validated or unvalidated version of the NDI was used. Moreover, responsiveness was assessed differently in this study since the analysis was performed in students rather than neck pain patients and participants were not treated between repeated measurements.

This study has several limitations. First, the sample was not recruited specifically for the purpose of validating the Neck Disability Index but was drawn from a number of randomized trials. All available patients from the respective trials were included in the analysis. Thus, no a priori sample size calculation was performed. However, the achieved sample was considerably larger than that of most other cross-cultural adaptations of the NDI [7-9,11,32,36,40]. Secondly, the sample comprised mainly female patients with low to moderate disability due to chronic non-specific neck pain. The psychometric properties found in this analysis might therefore not be fully applicable to patients with more severe neck pain and/or Whiplash-associated disorder. However, despite its limitations, this study comprises a large homogeneous sample to evaluate the reliability and validity of the German version of the Neck Disability Index.

## Conclusions

Comparable to the original English version, the German version of the Neck Disability Index demonstrated a single factor structure. The instrument has acceptable to good psychometric properties and is associated with other measures of health. The instrument further is sensitive to change after physical activity. As the original instrument, the German version of the Neck Disability Index seems to present a useful measure of neck disability in patients with chronic non-specific neck pain.

## Competing interest

The authors declare that there are no financial or non-financial conflicts of interest.

## Authors’ contributions

HC was responsible for conception and design of the study, analysis and interpretation of data, participated in acquisition of data, and drafted the manuscript. RL participated in conception and design of the study, acquisition of data, analysis and interpretation of data, and critically revised the manuscript. JL and GD participated in conception and design of the study, and critically revised the manuscript. AM participated in conception and design of the study, acquisition of data, and critically revised the manuscript. All authors read and approved the final manuscript.

## Pre-publication history

The pre-publication history for this paper can be accessed here:

http://www.biomedcentral.com/1471-2474/15/91/prepub

## Supplementary Material

Additional file 1 German version of the Neck Disability Index (NDI). Click here for file
