# Supplementary material for: Validation of the German version of the Neck Disability Index (NDI)
Source: BMC Musculoskelet Disord. 2014 Mar 19;15:91. doi: 10.1186/1471-2474-15-91 (PMC3999938; doi:10.1186/1471-2474-15-91)
Supplement: Additional file 1 — German version of the Neck Disability Index (NDI). [file 1471-2474-15-91-S1.pdf]

VP: ______ Datum: _________

Neck Disability Index

Dieser Fragebogen wurde entworfen, um Ihnen die Möglichkeit zu geben zu beschreiben, inwieweit ***Ihre Nackenschmerzen Ihren Alltag beeinträchtigen***. Bitte beantworten Sie jeden Abschnitt und kreuzen Sie für jeden der folgenden Abschnitte das Kästchen der Antwortmöglichkeit an, die ***am besten auf Sie zutrifft***. Auch wenn sich mehrere Sätze in einem Abschnitt auf Ihre Situation beziehen sollten, wählen Sie bitte den einen Satz aus, der Ihre Situation am ehesten beschreibt.

Schmerzintensität

O Ich habe im Moment keine Schmerzen.
O Ich habe im Moment sehr geringe Schmerzen.
O Ich habe im Moment mäßige Schmerzen.
O Ich habe im Moment ziemlich starke Schmerzen.
O Ich habe im Moment sehr starke Schmerzen.
O Ich habe im Moment die stärksten Schmerzen, die ich mir vorstellen kann.

Persönliche Körperpflege (Waschen, Ankleiden, etc.)

O Ich kann meine Körperpflege erledigen, ohne dass dies zusätzliche Schmerzen verursacht.
O Ich kann meine Körperpflege erledigen, aber dies verursacht mir zusätzliche Schmerzen.
O Das Erledigen der Körperpflege ist schmerzhaft, und ich bin dabei langsam und vorsichtig.
O Ich brauche etwas Hilfe, aber ich kann den größten Teil meiner Körperpflege selbst besorgen.
O Ich brauche täglich Hilfe bei den meisten Verrichtungen der Körperpflege.
O Ich ziehe mich nicht an, wasche mich nur mit Mühe und bleibe im Bett.

Heben

O Ich kann schwere Gegenstände ohne zusätzliche Schmerzen heben.
O Ich kann schwere Gegenstände heben, aber dies verursacht zusätzliche Schmerzen.
O Meine Schmerzen hindern mich daran, schwere Gegenstände vom Boden aufzuheben. Aber ich kann schwere Gegenstände heben, wenn sie günstig positioniert sind (z.B. auf dem Tisch).
O Meine Schmerzen hindern mich daran, schwere Gegenstände zu heben. Aber ich kann leichte bis mittelschwere Gegenstände heben, wenn sie günstig positioniert sind.
O Ich kann nur sehr leichte Gegenstände heben.
O Ich kann überhaupt nichts heben oder tragen.

Lesen

O Ich kann lesen, soviel ich will, ohne Nackenschmerzen zu bekommen.
O Ich kann lesen, soviel ich will, aber ich bekomme davon leichte Nackenschmerzen.
O Ich kann lesen, soviel ich will, aber ich bekomme davon mäßige Nackenschmerzen.
O Ich kann wegen mäßiger Nackenschmerzen nicht soviel lesen, wie ich will.
O Ich kann wegen starker Nackenschmerzen kaum lesen.
O Ich kann überhaupt nicht mehr lesen.

Kopfschmerzen

O Ich habe überhaupt keine Kopfschmerzen.
O Ich habe leichte Kopfschmerzen, die unregelmäßig auftreten.
O Ich habe mäßige Kopfschmerzen, die unregelmäßig auftreten.
O Ich habe mäßige Kopfschmerzen, die regelmäßig auftreten.
O Ich habe starke Kopfschmerzen, die regelmäßig auftreten.
O Ich habe die meiste Zeit Kopfschmerzen.

Konzentration

O Ich kann mich, wenn ich will, ohne Schwierigkeiten voll konzentrieren.
O Ich kann mich, wenn ich will, mit leichten Schwierigkeiten voll konzentrieren.
O Ich habe ziemliche Schwierigkeiten mich zu konzentrieren, wenn ich es will.
O Ich habe große Schwierigkeiten mich zu konzentrieren, wenn ich es will.
O Ich habe sehr große Schwierigkeiten mich zu konzentrieren, wenn ich es will.
O Ich kann mich überhaupt nicht konzentrieren.

Arbeit

O Ich kann soviel Arbeit erledigen, wie ich möchte.
O Ich kann nur meine übliche Arbeit erledigen, aber nicht mehr.
O Ich kann den größten Teil meiner üblichen Arbeit erledigen, aber nicht mehr.
O Ich kann meine übliche Arbeit nicht erledigen
O Ich kann kaum eine Arbeit erledigen.
O Ich kann überhaupt keine Arbeit erledigen.

Autofahren

O Ich kann Auto fahren, ohne Nackenschmerzen zu bekommen.
O Ich kann Auto fahren, solange ich will, mit leichten Nackenschmerzen.
O Ich kann Auto fahren, solange ich will, mit mäßigen Nackenschmerzen.
O Ich kann wegen mäßiger Nackenschmerzen nicht Auto fahren, solange ich will.
O Ich kann wegen starker Nackenschmerzen kaum Auto fahren.
O Ich kann überhaupt nicht Auto fahren.

Schlafen

O Ich habe keine Schlafprobleme.
O Mein Schlaf ist kaum gestört (weniger als 1 Stunde schlaflos).
O Mein Schlaf ist leicht gestört (1-2 Stunden schlaflos).
O Mein Schlaf ist mäßig gestört (2-3 Stunden schlaflos).
O Mein Schlaf ist stark gestört (3-5 Stunden schlaflos).
O Mein Schlaf ist komplett gestört (5-7 Stunden schlaflos).

Freizeit

O Ich kann alle meine Freizeitaktivitäten ohne Nackenschmerzen ausüben.
O Ich kann, wenn auch mit einigen Nackenschmerzen, alle meine Freizeitaktivitäten ausüben.
O Wegen Nackenschmerzen kann ich die meisten, aber nicht alle meiner üblichen Freizeitaktivitäten ausüben.
O Ich kann wegen Nackenschmerzen nur einige meiner Freizeitaktivitäten ausüben.
O Ich kann wegen Nackenschmerzen kaum irgendwelche Freizeitaktivitäten ausüben.
O Ich kann überhaupt keine Freizeitaktivitäten ausüben.
